# A Web-Based Antiretroviral Therapy Adherence Intervention (Thrive With Me) in a Community-Recruited Sample of Sexual Minority Men Living With HIV: Results of a Randomized Controlled Study

**DOI:** 10.2196/53819

**Published:** 2024-09-30

**Authors:** Keith J Horvath, Sara Lammert, Darin Erickson, K Rivet Amico, Ali J Talan, Ore Shalhav, Christina J Sun, H Jonathon Rendina

**Affiliations:** 1 Department of Psychology San Diego State University San Deigo, CA United States; 2 Division of Epidemiology and Community Health School of Public Health University of Minnesota Minneapolis, MN United States; 3 Health Behavior and Health Education School of Public Health University of Michigan Ann Arbor, MI United States; 4 Whitman-Walker Institute Washington, DC, MD United States; 5 City University of New York - Hunter College New York City, NY United States; 6 College of Nursing University of Colorado Anschutz Medical Campus Aurora, CO United States; 7 Department of Epidemiology Milken Institute School of Public Health George Washington University Washington DC, MD United States

**Keywords:** HIV, antiretroviral therapy, ART, mobile health, mHealth, intervention, men who have sex with men

## Abstract

**Background:**

Most new HIV infections are attributed to male-to-male sexual contact in the United States. However, only two-thirds of sexual minority men living with HIV achieve an undetectable viral load (UVL). We tested a web-based antiretroviral therapy adherence intervention called Thrive with Me (TWM) with core features that included medication self-monitoring and feedback, HIV and antiretroviral therapy information, and a peer-to-peer exchange.

**Objective:**

We assessed the efficacy of TWM on HIV UVL among adult (aged ≥18 years) sexual minority men. Moreover, we assessed the impact of overall engagement and engagement with specific intervention features on HIV UVL.

**Methods:**

In total, 401 sexual minority men (mean age 39.1, SD 10.8 y; 230/384, 59.9% African American) in New York City were recruited between October 2016 and December 2019 and randomized to receive TWM (intervention) or a weekly email newsletter (control) for 5 months. Computerized assessments occurred at baseline and months 5, 11, and 17. The primary outcome was a dichotomous measure of HIV UVL (≤20 copies/μL). Generalized estimating equations with robust SEs were used to assess the effect of the TWM intervention on HIV UVL over the follow-up period in an unadjusted model and a model adjusted for baseline differences and then stratified by baseline recent drug use urinalysis. In secondary analyses, generalized linear models were used to estimate risk differences in the association of overall engagement with TWM (the sum of the number of days participants accessed ≥1 screen of the TWM intervention out of a possible 150 days) and engagement with specific TWM components on HIV UVL throughout the 17-month intervention period.

**Results:**

Participant retention was 88.5% (355/401; month 5), 81.8% (328/401; month 11), and 80.3% (322/401; month 17). No consistent differences in HIV UVL were found between those randomized to receive TWM or the control at the 5- (difference-in-differences [DD]=–7.8, 95% CI –21.1 to 5.5), 11- (DD=–13.9, 95% CI –27.7 to 0.04), or 17-month (DD=–8.2, 95% CI –22.0 to 5.7) time points, or when stratified by baseline recent drug use. However, those TWM-assigned participants with high overall levels of engagement (in the upper 25th percentile) were more likely to have an HIV UVL at the end of the 5-month active intervention period compared to those with low engagement (below the 75th percentile; risk difference=17.8, 95% CI 2.5-33.0) or no engagement (risk difference=19.4, 95% CI 3.3-35.5) in the intervention. Moreover, high engagement with the peer-to-peer exchange was associated with HIV UVL over time in unadjusted models.

**Conclusions:**

TWM did not have overall impacts on HIV UVL; however, it may assist some sexual minority men who are highly engaged with this web-based intervention in achieving HIV viral suppression.

**Trial Registration:**

ClinicalTrials.gov NCT02704208; https://clinicaltrials.gov/study/NCT02704208

## Introduction

### Background

Sexual minority men, especially Black and Latinx men, are at highest risk of acquiring HIV in the United States [1]. It is estimated that 71% of all cases of HIV in the United States (and 89% among male individuals) in 2020 were attributed to male-to-male sexual contact, most of which occurred among Black (39%) and Hispanic or Latino (31%) sexual minority men [1]. Disparities persist in HIV treatment outcomes. Among people living with HIV, it is clear that greater efforts are needed to reach the Ending the HIV Epidemic initiative goal of 90% of people living with HIV being virally suppressed by 2030 [2]. High proportions of people living with HIV achieving an undetectable viral load (UVL) is important to both optimize individual health and substantially reduce onward transmission [3-5]. However, among male individuals living with HIV attributed to male-to-male sexual contact in 2020, a total of 82% were linked to care within 1 month of their diagnosis, 76% received any HIV medical care, and 67% achieved viral suppression in 2020 [6]. For these reasons, there is a continuing need for innovative antiretroviral therapy (ART) adherence interventions for sexual minority men living with HIV.

Sexual minority men living with HIV must navigate unique individual, social, and structural challenges—including intersectional stigma, racism, and economic and legal hardships—throughout the HIV continuum of care [7]. Drug use, particularly stimulant use [8], has been consistently associated with HIV seroconversion [9,10], as well as poorer ART adherence that leads to suboptimal rates of viral suppression [11-14]. Stimulant-using sexual minority men have greater difficulties achieving and sustaining viral suppression, which could unnecessarily quicken clinical HIV progression [15-17] and increase risk of onward HIV transmission [18-20].

Efforts to develop and test eHealth and, more narrowly, mobile health (mHealth) interventions to address the HIV prevention and care continuum have expanded in recent years [21-23]. Reviews of eHealth- and mHealth-based ART adherence interventions have found that most have pilot or quasi-experimental designs, and the results of these studies suggest a high potential of eHealth and mHealth approaches to improve ART adherence [21,22]. However, the results of larger randomized controlled trials of technology-based ART adherence interventions are mixed. One study showed significant reductions in plasma HIV viral load (VL) in the treatment arm compared to the control arm [24]. Other studies have shown impacts on electronic dose monitoring or self-reported adherence outcomes but not on plasma HIV VL [25-27] or only on plasma HIV VL for a subset of participants [28] or no impacts on plasma HIV VL or adherence [29]. For these reasons, further efforts are needed to understand how eHealth or mHealth interventions can be optimized to have impacts on clinically meaningful outcomes.

In addition to assessing overall intervention outcomes, it is critical that we have a better understanding of the effects of engagement with digital interventions on treatment outcomes [30]. Intervention engagement metrics (eg, the amount, frequency, duration, and depth of engagement with the entire or parts of a digital intervention) through the collection of paradata (ie, data collected on users’ actual activity in a digital intervention) may provide a more nuanced understanding of what features work for whom to advance digital intervention science [30-32]. A recent review of the use and findings of digital HIV prevention and treatment interventions for sexual and gender minority persons from 2017 to 2023 showed that, of the 33 digital HIV interventions published in those years, 19 reported paradata engagement metrics. However, only 6 studies reported the associations between intervention engagement and intermediate or primary study outcomes [30], of which 4 showed a positive association between intervention engagement (defined in a variety of ways) and health promotion behaviors [30]. Although these findings are encouraging, more information across a broad spectrum of digital HIV prevention and treatment interventions is needed to guide further investments into these approaches.

### Objectives

In this paper, we report the results of the Thrive with Me (TWM) web-based intervention [33] in a large community sample of sexual minority men living with HIV. We hypothesized that a higher proportion of participants in the TWM intervention than control participants would have HIV UVL after the intervention and that participants in the TWM intervention who had recently used illicit drugs would demonstrate the greatest improvements in HIV UVL after the intervention compared to participants who had not recently used illicit drugs. As a secondary aim, we examined the effect of level of engagement with the TWM intervention and its unique components on HIV UVL among those assigned to the TWM study arm. Such an analysis, although infrequently done [30,31], is critical to increase understanding of the degree to which exposure to eHealth or mHealth interventions and their individual components may be associated with HIV outcomes.

## Methods

### Participants and Procedures

Sexual minority men residing in the New York City metropolitan area were recruited between October 2016 and April 2018 by staff at a lesbian, gay, bisexual, transgender, and queer health research center at Hunter College, City University of New York. Interested persons completed a web-based screening survey that assessed eligibility across multiple studies; next, persons who met the basic criteria (eg, living with HIV) for TWM were contacted to complete a second screener specific to the TWM study to determine eligibility. Eligibility criteria were (1) identified current gender as male, (2) report of having had sex with a man in the previous year, (3) HIV-positive serostatus, (4) self-report of a detectable VL in the previous year or ART adherence of <90% in the previous 30 days, (5) English-language proficiency, (6) the ability to send or receive SMS text messages, and (7) internet access over the course of the active intervention (5 months). Recruitment included a 50% target of self-reported street drug use (ie, powder cocaine, crack cocaine, painkillers, methamphetamine, heroin, hallucinogens, recreationally used prescription drugs, ketamine, methylenedioxymethamphetamine, and poppers). Participants meeting the inclusion criteria were invited to attend an in-person enrollment visit that included completing a consent process with web-based documentation of consent, a computerized survey, urine-based drug use test, and a blood draw to assess HIV VL at the research offices at baseline and months 5 (immediately after the intervention), 11, and 17.

### Randomization and Blinding

Following consent and completion of the enrollment activities described previously, research staff used a computer algorithm to randomize participants to either the TWM arm or control arm (1:1) using blocks of 20 (10 for the intervention and 10 for the control group). The same computer algorithm stratified enrollment by recent drug use (ie, reporting use of powder cocaine, crack cocaine, painkillers, methamphetamine, heroin, hallucinogens, prescription drugs used recreationally, ketamine, methylenedioxymethamphetamine, or poppers in the previous 30 days) and nonrecent drug use based on their self-reported substance use in the baseline survey. Study arms were described to participants as “Group 1” and “Group 2”; however, study staff were not blinded as to which arm the participants were assigned to.

### The TWM and Control Groups

Participants were onboarded to either the TWM or control condition as the final step during the enrollment visit. Participants randomized to the TWM study arm created a username and password that would give them free access to the intervention, and study staff provided information about each part of the intervention, described in the following paragraph. Participants randomized to the control condition were told that they should receive the first email with the newsletter that week.

TWM is a 5-month (150 days) mobile-optimized web-based intervention to improve ART adherence among adult (aged ≥18 years) HIV-positive sexual minority men residing in New York City with detectable VL or problematic ART adherence at baseline. An earlier pilot trial of TWM demonstrated feasibility, acceptability, and preliminary impact [34]. TWM is grounded in the Information-Motivation-Behavioral Skills (IMB) model [35,36] and has the following core components: (1) a peer exchange (similar to Facebook) that allows participants to post and comment on text, pictures, and videos in an unstructured format; (2) brief, tailored HIV and ART adherence informational articles (called Thrive Tips; 300 were created in total) that were delivered daily, with the ability to search past content; (3) daily SMS text message–based ART reminders that also provide an option for participants to report (through texting back) their ART adherence and overall mood for that day; and (4) weekly reflection on adherence, mood, and substance use. In addition, the TWM site has a profile page to update settings and viewpoints and badges earned, an “about us” page, and pages to explain how to use the site. Participants randomized to the TWM intervention were encouraged during the enrollment visit to use the intervention regularly; however, they were not required to meet a minimum engagement standard. A weekly draw for a US $25 gift card for those who had used the site ≥5 times in the previous 10 days was held during the active study period.

Participants randomized to the control condition received a weekly email for 21 consecutive weeks that contained a link that, when clicked on, opened a web page with information on a specific topic related to living with HIV and improving general well-being (eg, HIV disclosure and managing stress) but not specifically about ways to improve ART adherence. An example weekly newsletter on the topic of managing stress is shown in Multimedia Appendix 1. More information about the TWM intervention and control arms is available elsewhere [33].

### Measures

#### Laboratory Measures

##### Plasma HIV-1 VL

Blood draws were collected at baseline and the 5-, 11-, and 17-month follow-ups at the Hunter research offices by a trained phlebotomist and assayed by Quest Diagnostics for the presence of plasma HIV-1 RNA. To assess the primary outcome, we used anything below the lower limit of detection of 20 copies per microliter to define HIV UVL.

##### Drug Use

Participants completed urine screens at each assessment time point using the Integrated E-Z Split Key Cup II-5 panel (Innovacon Laboratories) to detect the following substances: tetrahydrocannabinol (THC; marijuana), methamphetamine, amphetamine, cocaine, and opioids. This test can detect methamphetamine, amphetamine, cocaine, and opioids from 1 to 4 days after use and THC (marijuana) for up to 30 days after use. For this analysis, a positive baseline urinalysis was defined as any detectable measure of methamphetamine, amphetamine, cocaine, or opioids.

#### Survey Measures

##### Sociodemographic Characteristics

At baseline, participants were asked common sociodemographic factors, including age (in years), race and ethnicity, highest level of education, and current employment status. Additional HIV history data were collected, including 30-day ART adherence.

##### Psychosocial Characteristics

The following psychosocial characteristics were assessed using a computerized survey at each assessment point, of which baseline measures were used for the purpose of characterizing participants in this study. Depressive symptoms were assessed using the 10-item Center for Epidemiologic Studies Depression Scale [37] and dichotomized into depressive symptoms (score of ≥10) and no depressive symptoms (score of <10). ART-related information (9 items), personal and social motivation (10 items), and behavioral skills (14 items) were assessed using the IMB ART Adherence Questionnaire [38]. HIV stigma, including internalized (6 items), anticipated (9 items), and enacted (9 items) stigma was assessed using the HIV Stigma Scale [39]. Social support, including emotional or information support (8 items), tangible support (4 items), affectionate support (3 items), positive social interaction (3 items), and an overall social support score, was assessed using the Medical Outcome Study Social Support Survey [40]. Life chaos and perceived stress were assessed using the 6-item Life Chaos Scale [41] and the 14-item Perceived Stress Scale [42], respectively. Finally, alcohol use was assessed using the Alcohol Use Disorders Identification Test [43].

#### TWM Intervention Engagement Measures

##### Overview

TWM engagement data were only collected from individuals randomized to receive the TWM intervention (202/401, 50.4%) during the 5-month (150 days) active intervention period. Engagement data were collected via a customized back-end website accessible only to study staff and investigators that provided these data in CSV files that were updated as participants engaged with the intervention. These CSV files were downloaded after study participants were no longer active in the TWM intervention arm and cleaned for analyses.

##### Overall TWM Engagement

To quantify overall engagement with the TWM intervention, we summed the number of days on which a participant accessed at least one screen of the TWM intervention out of a possible 150 days. Those who did not log onto the TWM intervention during the intervention period were categorized as nonengagers, whereas those who accessed at least one screen of the TWM intervention were considered engagers. Next, those who engaged with TWM were further categorized as having high or low engagement based on dichotomization at the 75th percentile. While there are no established thresholds for definitions of engagement with digital interventions, we chose this level of engagement (as opposed to other possible options, such as the 50th percentile) to represent participants who were more obviously active in the TWM intervention than, for example, a participant who was above the 50th percentile but not by much (eg, the 55th percentile). Using this cutoff, engagement with TWM was categorized as follows: nonengagers (0 days active), low engagement with TWM (1-33 days active), and high engagement with TWM (≥34 active days).

##### Engagement With Individual TWM Components

We calculated engagement with three individual TWM components: (1) peer exchanges, (2) Thrive Tips (ie, brief informational pieces of content), (3) and ART adherence self-monitoring. Engagement with the asynchronous peer exchanges was measured using the sum of the number of unique wall posts and comments made by a user during the TWM intervention period (ie, up to 150 days). Engagement with the Thrive Tips was calculated as the number of unique Thrive Tips that participants viewed (possible range 0-300). Engagement with the ART adherence self-monitoring feature was the number of days on which participants reported their ART adherence (possible range 0-150). Participants were categorized as having high or low engagement with the individual TWM components based on dichotomization of each component at the 75th percentile.

### Statistical Analyses

Descriptive statistics (means and SDs and frequencies and percentages) were generated to describe the participants in the TWM study. Appropriate statistical tests were used to compare numeric (2-tailed *t* test) and categorical (chi-square test) variables between the TWM intervention and control arms at baseline. All analyses were conducted using Stata (version 14; StataCorp), and significance was established at *P*<.05.

To account for missing data during follow-up, we used multiple imputation. Demographic, psychosocial, and substance use factors that were associated with having an HIV UVL (<20 copies/μL) at baseline were used to impute missing variables at subsequent time points and HIV UVLs at the 5-, 11-, and 17-month follow-ups.

We used generalized estimating equations with robust SEs to assess the effect of the TWM intervention on HIV UVL over the follow-up period in an unadjusted model and a model adjusted for baseline differences and then stratified by baseline urinalysis (ie, positive vs negative drug use). The main effects of interest were the condition-by-time interaction terms at months 5, 11, and 17. The Stata *xtgee* command was used to calculate the difference-in-differences (DD) estimate as the proportion of those with an HIV UVL between participants in the TWM intervention arm and those in the control arm. Described in more detail elsewhere [44], a DD estimation first calculates the difference between each follow-up time point and baseline estimates within each study arm; using those within-arm difference estimates, the DD estimate (and the associated CI) is arrived at by finding the difference between study arms for each assessment time point.

To assess the impact of engagement with the TWM intervention on viral suppression, we modeled overall engagement with the TWM intervention as well as engagement with the 3 TWM components described previously on HIV UVL (<20 copies/μL) throughout the 17 months for participants assigned to the TWM intervention (202/401, 50.4%). Generalized linear models with robust SEs were used to estimate risk differences (RDs) and 95% CIs of the association of overall engagement with TWM and engagement with specific TWM components on viral suppression throughout the 17-month intervention period, including unadjusted models and models adjusted for differences between TWM users and nonusers.

### Ethical Considerations

All study procedures were approved by the University of Minnesota Institutional Review Board and Hunter College, City University of New York, Institutional Review Board (IRB Study 1120). In addition, a Data Safety and Monitoring Board was established to provide regular oversight of research practices and activities to protect human participants and the integrity of the data in the study. Written informed consent was provided by all study participants, and data were deidentified before analyses for this study. Participants were paid US $50 in cash at the baseline and 5-, 11-, and 17-month assessments. Participants in the intervention group were also eligible to win a weekly prize draw for a US $25 electronic gift card if they were active on the TWM website for 5 out of the 10 preceding days. This study was registered on the national registry of clinical trials at ClinicalTrials.gov (NCT02704208).

## Results

**Figure 1:**
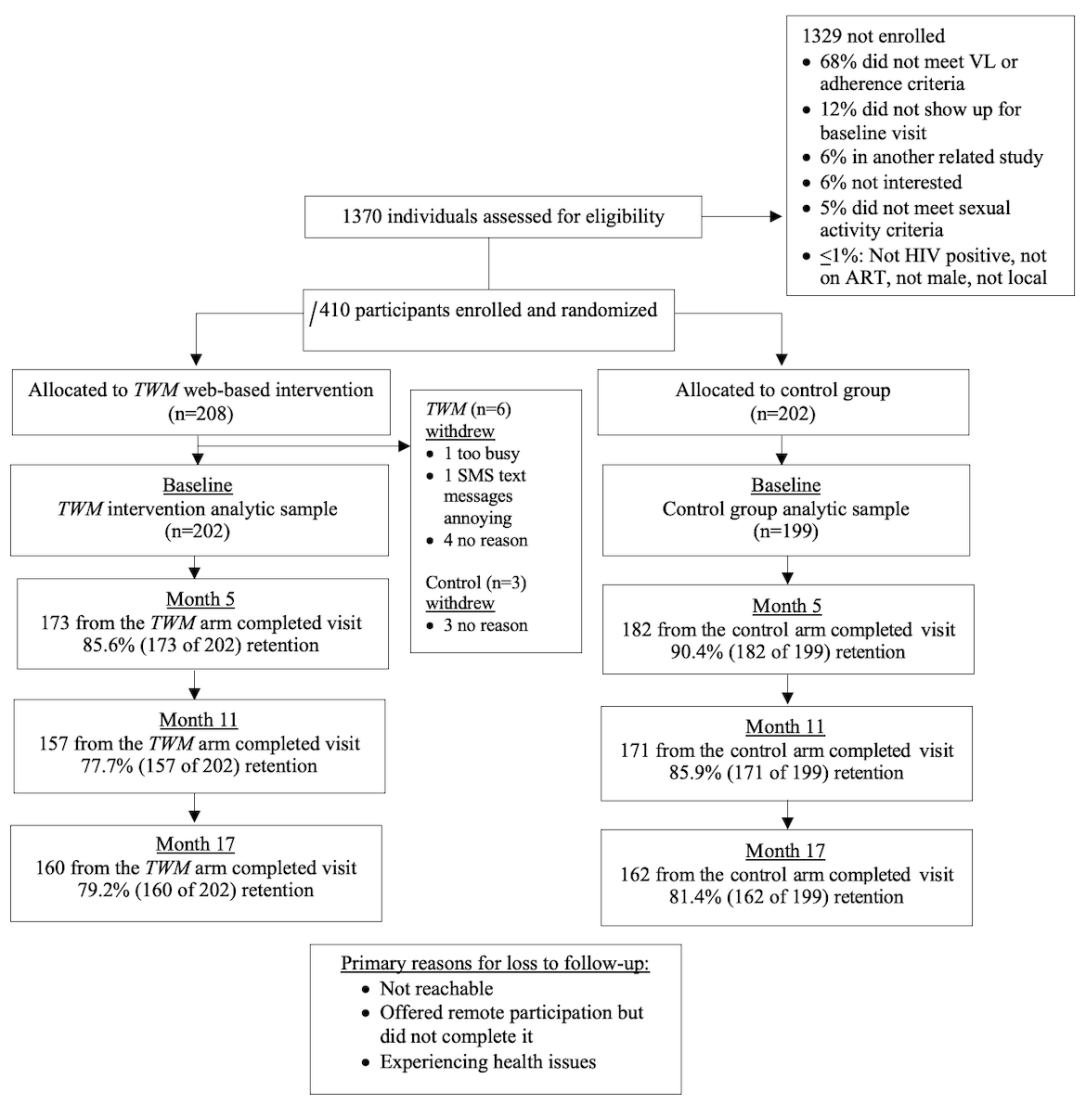
Thrive with Me (TWM) intervention trial CONSORT (Consolidated Standards of Reporting Trials) diagram. ART: antiretroviral therapy; mHealth: mobile health; VL: viral load.

A total of 401 men who have sex with men living with HIV were recruited to participate in the TWM study. Following a 1:1 randomization, 50.4% (202/401) of the participants were randomized to receive the TWM intervention, whereas 49.6% (199/401) were randomized to receive the control condition (Figure 1).

### Baseline Characteristics

Table 1 shows participant characteristics for the full sample and by study arm. The average age of the participants was 39.1 (SD 10.8) years. More than half (230/384, 59.9%) of the participants identified as African American or Black individuals, 29.4% (113/384) identified as White individuals, and almost 8% (31/384, 8.1%) identified as more than one race. In addition, more than a quarter (108/401, 26.9%) identified as Hispanic or Latino. More than 40% (175/397, 44.1%) were employed full time or part time, and an additional 39.3% (156/397) were unemployed. Overall, approximately three-quarters of the participants (303/400, 75.8%) had some college education or a college degree, with a higher proportion of participants in the control than the TWM arm having a college degree (84/199, 42.2% vs 52/201, 25.9%, respectively). Nearly half (194/401, 48.4%) of the participants self-reported depressive symptoms at baseline. No group differences were found for perceived stress, HIV stigma, social support, or life chaos.

Excluding marijuana, approximately one-quarter of the participants (113/401, 28.2%) at baseline had detectable levels of substance use from urinalysis. Over 10% of the sample exhibited baseline levels of methamphetamine (58/401, 14.5%), cocaine (52/401, 13%), and amphetamine (45/401, 11.2%); however, only 1.2% (5/401) of the participants tested positive for opioids. A total of 41.1% (165/401) of the participants tested positive for THC at baseline. More than one-quarter of the participants (116/401, 28.9%) self-reported hazardous or harmful alcohol use or alcohol dependence.

At baseline, participants reported an average percentage of ART adherence in the previous 30 days of 87.6% (SD 17.6%), with no group differences found in this or the IMB ART Adherence Questionnaire ART-related information, motivation, or behavioral skills variables.

**Table 1:** Baseline characteristics of the participants in the Thrive with Me (TWM) intervention and control conditions.

				Total (N=401)		TWM study arm (n=202)		Control study arm (n=199)
**Demographics**								
	Age (y), mean (SD)		39.1 (10.8)		40.1 (10.8)		38.1 (10.6)	
	**Race, n (%)^a^**							
		African American or Black	230 (59.9)		123 (63.7)		107 (56)	
		American Indian or Alaska Native	5 (1.3)		1 (0.5)		4 (2.1)	
		Asian	3 (0.8)		1 (0.5)		2 (1)	
		Native Hawaiian or Pacific Islander	2 (0.5)		2 (1)		0 (0)	
		White	113 (29.4)		54 (28)		59 (30.9)	
		>1 Race	31 (8.1)		12 (6.2)		19 (9.9)	
	Hispanic, n (%)		108 (26.9)		62 (30.7)		46 (23.1)	
	**Educational level, n (%)^b^**							
		High school or lower	97 (24.3)		59 (29.4)		38 (19.1)	
		Some college education, associate degree, and technical degree	167 (41.8)		90 (44.8)		77 (38.7)	
		College, postgraduate, and professional degree	136 (34)		52 (25.9)		84 (42.2)	
	**Employment status, n (%)^c^**							
		Full time	87 (21.9)		41 (20.5)		46 (23.4)	
		Part time	88 (22.2)		45 (22.5)		43 (21.8)	
		Unemployed	156 (39.3)		77 (38.5)		79 (40.1)	
		Disabled	63 (15.9)		35 (17.5)		28 (14.2)	
		Retired	3 (0.8)		2 (0.1)		1 (0.5)	
**VL^d^ and ART^e^ adherence measures**								
	**VL (≤20 copies/μL), n (%)^f^**							
		Detectable VL	154 (38.5)		74 (36.8)		80 (40.2)	
		Undetectable VL	246 (61.5)		127 (63.2)		119 (59.8)	
	30-day adherence, mean (SD)		87.6 (17.6)		87.2 (18.6)		88.0 (16.6)	
	**IMB^g^ scale for ART adherence, mean (SD)**							
		Information	36.7 (5.8)		37.0 (5.8)		36.4 (5.9)	
		Motivation	34.0 (8.4)		34.8 (8.1)		33.2 (8.5)	
		Behavior	48.2 (8.5)		48.8 (8.3)		47.6 (8.8)	
**Drug and alcohol use measures**								
	**Positive urinalysis^h^, n (%)**		113 (28.2)		65 (32.2)		48 (24.1)	
		Marijuana use	165 (41.1)		88 (43.6)		77 (38.7)	
		Methamphetamine use	58 (14.5)		31 (15.3)		27 (13.6)	
		Amphetamine use	45 (11.2)		25 (12.4)		20 (10.1)	
		Cocaine use	52 (13)		33 (16.3)		19 (9.5)	
		Opioid use	5 (1.2)		2 (1)		3 (1.5)	
	Hazardous or harmful alcohol use, n (%)		116 (28.9)		61 (30.2)		55 (27.6)	
**Additional sociodemographics**								
	Depressive symptoms, n (%)		194 (48.4)		100 (49.5)		94 (47.2)	
	Perceived Stress Scale score, mean (SD)		16.8 (7.3)		17.0 (7.3)		16.6 (7.4)	
	**HIV Stigma Scale score, mean (SD)**							
		Internalized stigma	2.2 (1.1)		2.2 (1.1)		2.2 (1.1)	
		Anticipated stigma	2.0 (0.9)		2.0 (0.9)		2.0 (0.8)	
		Enacted stigma	1.5 (0.7)		1.5 (0.7)		1.6 (0.7)	
	**Medical Outcome Study Social Support Survey score, mean (SD)**							
		Emotional support	3.5 (1.2)		3.6 (1.2)		3.5 (1.2)	
		Affectionate support	3.5 (1.3)		3.5 (1.3)		3.4 (1.4)	
		Tangible support	3.1 (1.4)		3.1 (1.4)		3.1 (1.4)	
		Social interaction support	3.6 (1.3)		3.6 (1.2)		3.5 (1.3)	
		Overall social support	3.4 (1.1)		3.5 (1.1)		3.4 (1.2)	
	HIV life chaos (Life Chaos Scale), mean (SD)		15.9 (4.8)		15.8 (4.7)		15.9 (4.8)	

^a^17 participants did not report race.

^b^1 participant did not report educational level.

^c^4 participants did not report employment status.

^d^VL: viral load.

^e^ART: antiretroviral therapy.

^f^1 participant did not undergo VL testing.

^g^IMB: Information-Motivation–Behavioral Skills model.

^h^A positive urinalysis was defined as any detectable measure of methamphetamine, amphetamine, cocaine, or opioids.

### Study Retention

Overall, nearly 90% of the participants were retained at month 5 (355/401, 88.5%), whereas 81.8% (328/401) and 80.3% (322/401) were retained at months 11 and 17, respectively. Differences in retention were found at month 11 (intervention: 157/202, 77.7% vs control: 171/199, 85.9%). On average, those who were retained across both study arms at month 11 had lower affectionate support (mean: 3.4; SD: 1.4) compared to those who were not retained (mean: 3.8; SD: 1.3). No other sociodemographic differences by study arm were found. Retention by treatment and control group can be found in the CONSORT (Consolidated Standards of Reporting Trials) [45] flow diagram in Figure 1.

### The Effect of TWM on HIV VL for the Overall Sample and Stratified by Baseline Drug Use

Approximately two-thirds of the participants had an HIV UVL at baseline (266/401, 66.3% in the adjusted model), as shown in Table 2. The proportion of all participants with an HIV UVL remained relatively consistent over the follow-up period (month 5: 239/401, 59.6%; month 11: 224/401, 55.9%; month 17: 219/401, 54.5%).

Overall, the proportion of participants with HIV UVL decreased from baseline to month 17 across both study arms. In unadjusted models, there was no difference in HIV UVL between those randomized to receive the TWM intervention and those randomized to the control group at any of the follow-up time points, as shown in Table 3. Similarly, adjusting for baseline educational level, there was no difference in HIV UVL between those randomized to receive the TWM intervention and the control arm at the 5- and 17-month assessments. However, at the 11-month assessment, participants randomized to the TWM intervention were less likely to have an HIV UVL compared to those randomized to the control group in the adjusted model (DD=–13.9 95% CI –27.7 to –0.04), as shown in Table 3.

When stratified by baseline drug use, the proportion of participants with HIV UVL was higher among those with a negative urinalysis compared to those with a positive urinalysis at baseline. Among those with a positive urinalysis at baseline, individuals randomized to the TWM intervention were less likely only at the 11-month assessment to have an HIV UVL compared to those randomized to the control group in both unadjusted (DD=–26.8, 95% CI –49.9 to –3.6) and adjusted (DD=–27.2, 95% CI –52.5 to –1.9) models, as shown in Table 3. However, this difference was not found at any other time point for those with a positive urinalysis or at any time point for participants with a negative baseline urinalysis.

**Table 2:** The effect of Thrive with Me (TWM) on HIV-1 undetectable viral load (UVL) for the full sample and by study arm.

				Baseline UVL, estimate (95% CI)		Month 5 UVL, estimate (95% CI)		Month 11 UVL, estimate (95% CI)		Month 17 UVL, estimate (95% CI)
**TWM** **intervention (n=202)**										
	**Full sample**									
		Unadjusted	63.1 (56.4-59.7)		57.9 (50.7-65.0)		54.2 (46.9-61.6)		52.9 (45.2-60.6)	
		Adjusted	66.3 (59.8-72.7)		59.6 (52.4-66.8)		55.9 (48.5-63.3)		54.5 (46.8-62.2)	
	Positive drug urinalysis									
		Unadjusted	48.5 (36.2-60.7)		42.1 (29.3-54.8)		30.5 (17.5-43.5)		34.2 (21.0-47.3)	
		Adjusted	51.1 (39.0-63.2)		44.8 (31.6-58.1)		31.0 (17.5-44.6)		35.4 (21.6-49.2)	
	Negative drug urinalysis									
		Unadjusted	70.1 (62.4-77.7)		66.4 (58.2-74.6)		65.0 (56.2-73.8)		61.6 (52.8-70.5)	
		Adjusted	73.1 (65.7-80.4)		67.4 (59.4-75.5)		66.8 (58.2-75.3)		62.7 (54.0-71.5)	
**Control group (n=199)**										
	**Full sample**									
		Unadjusted	59.8 (53.0-66.6)		60.9 (53.9-67.9)		63.9 (56.5-71.2)		56.8 (49.2-64.5)	
		Adjusted	58.1 (51.6-64.7)		59.3 (52.6-65.9)		61.6 (54.6-68.7)		54.5 (47.1-62.0)	
	Positive drug urinalysis									
		Unadjusted	47.9 (33.8-62.0)		54.1 (38.9-69.3)		56.7 (42.2-71.1)		46.7 (30.5-62.8)	
		Adjusted	46.0 (32.9-59.1)		51.5 (37.8-65.2)		53.1 (39.8-66.4)		42.2 (27.4-57.0)	
	Negative drug urinalysis									
		Unadjusted	63.6 (55.9-71.3)		62.6 (54.7-70.6)		66.6 (58.3-74.8)		60.4 (51.6-69.2)	
		Adjusted	62.1 (54.6-69.6)		61.6 (53.8-69.3)		64.9 (56.9-72.9)		58.9 (50.3-67.6)	

**Table 3:** Difference-in-differences estimates of the effect of Thrive with Me (TWM) on HIV-1 undetectable viral load (UVL) for the full sample stratified by positive and negative drug urinalysis.

Difference-in-differences estimates^a^		Baseline to month 5, estimate (95% CI)	Baseline to month 11, estimate (95% CI)	Baseline to month 17, estimate (95% CI)
**Full sample (N=401)**				
	Unadjusted	–6.4 (–20.1 to 7.4)	–12.9 (–27.1 to 1.4)	–7.2 (–21.5 to 7.0)
	Adjusted	–7.8 (–21.1 to 5.5)	–13.9 (–27.7 to –0.04)	–8.2 (–22.0 to 5.7)
**Positive drug urinalysis**				
	Unadjusted	–12.5 (–34.7 to 9.6)	–26.8 (–49.9 to –3.6)	–13.1 (–38.5 to 12.4)
	Adjusted	–11.7 (–37.8 to 14.3)	–27.2 (–52.5 to –1.9)	–11.9 (–39.0 to 15.2)
**Negative drug urinalysis**				
	Unadjusted	–2.8 (–18.5 to 13.0)	–8.1 (24.4 to 8.3)	–5.3 (–21.6 to 11.1)
	Adjusted	–5.1 (–20.4 to 10.2)	–9.1 (–24.9 to 6.8)	–7.1 (–23.1 to 8.8)

^a^The between–study arm difference calculated from the difference between each follow-up assessment and baseline within study arms.

### The Effect of Engagement With TWM on HIV VL for Participants Randomized to the TWM Intervention

Of the 202 participants who were randomized to receive the TWM intervention, 110 (54.5%) accessed the TWM intervention at least once during the 5-month intervention (ie, engagers), whereas 92 (45.5%) did not use any of the TWM intervention components (ie, nonengagers). There were no demographic differences between TWM engagers and nonengagers. However, participants who used the TWM intervention were less likely to have a detectable VL (32/110, 29.1%) at baseline compared to those who did not use the intervention (42/92, 46%). TWM engagers were also less likely to have a positive urinalysis for methamphetamine (10/110, 9.1% vs 21/92, 23%) and amphetamines (6/110, 5.5% vs 19/92, 21%) compared to nonengagers.

Table 4 shows the median frequency and IQR for overall engagement and engagement with each of the 3 individual components (frequency of asynchronous peer exchanges, number of unique Thrive Tips viewed, and daily ART self-monitoring) by whether participants were considered low engagers (75th percentile) or high engagers (25th percentile) in each of the variables. With the exception of daily ART self-monitoring, substantial differences in engagement were found between low and high engagers. For example, low engagers had an average of only 7 active days in the intervention, whereas high engagers had an average of 78 active days. While differences in daily ART self-monitoring were evident among low and high engagers (100 days for low engagers vs 144 days for high engagers), they were not as stark as with other features.

**Table 4:** Frequency of Thrive with Me (TWM) user engagement among participants randomized to TWM and who accessed it at least once.

TWM user engagement		TWM-engaged participants (n=110), median (IQR)	Low engagers^a^, median (IQR)	High engagers^a^, median (IQR)
Overall engagement—number of active days		16 (5-33)	9 (4-20.5)^b^	77.5 (40-115)^c^
**Number of peer exchanges^d^**		7 (2-34)	4 (2-9)^e^	135 (58-195)^f^
	Wall posts	3 (1-13)	2 (1-5)^e^	34.5 (20-68)^f^
	Comments	3 (0-12)	1 (0-5)^e^	91 (29-142)^f^
Number of unique Thrive Tips accessed		6 (2-32)	4 (2-10)^g^	121 (78-168)^h^
Number of ART^i^ self-monitoring days		117 (51-137)	100 (41-124)^j^	144.5 (142-147)^c^

^a^Engagement dichotomized at the 75th percentile for each user engagement variable.

^b^n=84.

^c^n=26.

^d^Excluding 1 individual.

^e^n=83.

^f^n=27.

^g^n=75.

^h^n=25.

^i^ART: antiretroviral therapy.

^j^n=79.

Multimedia Appendix 2 shows the effect of overall engagement with TWM on HIV UVL, as well as the effect of engagement with the individual components of TWM on HIV UVL, at each assessment time point. At month 5 (the end of the intervention period), 73% (19/26) of TWM users with overall high engagement (ie, were in the 25th percentile of active intervention use days) were virally suppressed compared to 54% (45/84) of those categorized as having low engagement and 40% (37/92) of nonengagers. At month 5, high engagers were more likely to be virally suppressed compared to nonengagers in both the unadjusted (RD=31.9, 95% CI 12.6-51.3) and adjusted (RD=19.4, 95% CI 3.3-35.5) models. Similarly, high engagers were more likely to be virally suppressed compared to low engagers in the unadjusted (RD=23.4, 95% CI 4.3-42.4) and adjusted (RD=17.8, 95% CI 2.5-33.0) models (not shown in the table). However, no relationship was found between engagement categories and viral suppression at months 11 or 17.

Notably, 70% (19/27) of those categorized as having high engagement with peer exchanges (ie, were in the 25th percentile) were virally suppressed at month 5 compared to 54% (45/83) of those with low engagement and 40% (37/92) of nonengagers, as shown in Multimedia Appendix 2. High engagers with the peer exchange component were more likely to be virally suppressed than participants who did not engage with the peer exchanges at all at months 5 (RD=25.3, 95% CI 4.9-45.7), 11 (RD=22.5, 95% CI 1.1-44.0), and 17 (RD=23.4, 95% CI 2.2-44.7) in unadjusted models, although these effects were not found in adjusted models. We found no differences in viral suppression between engagement categories when examining the Thrive Tips or ART self-monitoring components at any assessment time point.

## Discussion

### Principal Findings

We did not find that the 5-month TWM web-based intervention delivered to a community-recruited sample of sexual minority men living with HIV overall improved their VL outcomes over 17 months, nor did we find that the intervention showed greater impact for participants with recent drug use at study entry. However, overall high engagement with the intervention compared to no engagement or low engagement among participants assigned to the TWM intervention arm was associated with having an HIV UVL at month 5, which was the active intervention use period (after which access to the intervention ended).

Behavioral ART adherence interventions have had mixed success in modifying adherence behaviors and reducing VL. In a meta-analysis of interventions cotargeting one or more syndemic factors (eg, mental health and substance use) and HIV prevention or treatment outcomes, significant effects were found for sexual risk reduction interventions but not for ART adherence interventions [46]. SMS text messaging and voice (eg, telephone-based) interventions have shown some success for improving adherence or viral suppression in lower-income countries [47]. However, mobile apps and computerized interventions most commonly used in higher-income countries, with the exception of a computerized intervention by Kurth et al [24], either lack sufficient power to be conclusive [47] or have not yet demonstrated effects on plasma HIV VL [25-29].

An important factor that may drive the degree to which digital ART adherence interventions are impactful is whether they are embedded in other aspects of HIV clinical care. TWM was designed and tested as a stand-alone ART adherence intervention that could supplement the HIV clinical care of sexual minority men living with HIV. In contrast, the computerized ART adherence intervention by Kurth et al [24] was conducted in 4 sessions during regular HIV clinic appointments and demonstrated positive impacts on HIV VL [24]. This arrangement may have provided a more successful context to engage participants with the intervention than mobile apps or computerized interventions that rely on participants engaging with them outside the clinic context. However, providing technology-based interventions in the clinic may diminish the potential benefits of these types of interventions, including lower implementation costs (ie, requiring less staff time), circumventing clinic-based stigma, and direct-to-consumer availability [21,48,49]. A better understanding of the trade-offs of the venues where technology-based interventions are deployed and the accompanying staffing requirements is needed.

While results did not support that the overall sample benefited from the intervention, those TWM-assigned participants with high levels of engagement (in the 25th percentile) were more likely to have an HIV UVL at the end of the 5-month active intervention period compared to those with low engagement (below the 75th percentile) or no engagement at all with the intervention. This finding is consistent with those of an ART adherence gaming intervention to improve rates of viral suppression among youth aged 16 to 24 years living with HIV called Epic Allies [29]. In that study, participants were enrolled primarily in HIV clinics that served a large number of youths, but engagement with the app occurred outside the clinic over 26 weeks. While no intervention effects on HIV VL were shown in an intention-to-treat analysis, youths who used the mobile app ≥4 days a week were 56% more likely to achieve viral suppression compared to youths who used the app less often at the 3-month assessment; however, those effects dissipated in later follow-up assessments. This and other studies showing that the impacts of mHealth interventions dissipate over time [50] suggest that optimizing technology-based ART adherence interventions will require robust management of participants to get and keep them engaged with the intervention.

Little is known about the impact of individual components of eHealth and mHealth interventions on HIV VL even though having a better understanding of which components may be the most impactful is needed [31]. A previous study by Bonett et al [51] found that engagement (measured as total time spent) with different features of the mHealth intervention among HIV-negative youth was associated with sociodemographic and behavioral factors (eg, participants who spent more time using the HIV and sexually transmitted infection testing site locator were more likely to be single). In the TWM arm of our study, we found high proportions of HIV UVL among users with high engagement with the peer exchange feature (both posting and commenting) in the unadjusted models at all follow-up time points, although not when adjusted for baseline viral suppression and positive drug urinalysis. Among high-engagement participants, all of them posted at least one wall comment, and 75% (20/27) of them responded to another participant’s post. The content of peer exchanges on social platforms focused on health promotion among people living with HIV may play a critical role. A previous qualitative analysis of the peer exchanges in TWM showed that social support was the most common theme from the analysis of peer exchanges, with half of social support exchanges coded as seeking social support and half as providing social support [52]. Peer exchanges also included issues regarding HIV treatment and care, such as challenges taking ART, adherence strategies, discussing HIV treatment including laboratory tests or side effects, and participants’ relationships with health care providers [52]. A recent study by Bauermeister et al [53] found that young (aged 18-30 years) Black sexual minority men who discussed experiences of stigma in a peer exchange forum during an mHealth intervention trial showed decreased anticipated stigma over time, whereas those whose discussions focused on sexuality-related stigma reported increased levels of internalized homophobia and sexual prejudice. Together, the results of the latter study and our TWM study suggest that a greater understanding of how peer exchanges may impact HIV care outcomes and other dimensions of well-being is warranted.

### Study Limitations

This study has important limitations that contextualize the findings. First, approximately 39% of the participants (154/401, 38.5%) entered the study with detectable HIV VL, which may have hindered our ability to demonstrate effects on VL as many participants started the study virally suppressed. As with many ART adherence studies, we balanced our ability to recruit our sample, who were required to self-report adherence problems, with more rigorous study inclusion (eg, detectable VL). Nonetheless, our findings suggest potential positive impact among those using the TWM intervention. Second, participants were recruited in the New York City area, which may have better technology infrastructure, higher technology adoption rates, and other unique considerations that differ from those in other regions of the United States. Therefore, the results may not generalize to other areas of the United States. Third, while our findings suggest a possible benefit of TWM for high-engaging participants, only approximately half (110/202, 54.5%) of those randomized to the TWM intervention actually engaged with it. We only incentivized intervention use through gift card draws, and therefore, this approach may mimic “real-world” use of this type of computerized intervention in the context of deploying minimal staffing to engage participants. Finally, improvement in VL may be due to an exogenous factor unrelated to study participation for men who chose to access TWM. For this reason, caution should be used when interpreting study findings for this group of men.

### Conclusions

Keeping limitations in mind, we believe that the results fill a critical gap in eHealth and mHealth ART adherence intervention science by adding to evidence that these types of interventions may need to be developed alongside in-person or internet-based support to have the greatest impacts on HIV VL. Using stepped-care models [54] to identify which participants need more intensive support holds promise in this area. Aside from using different intervention designs to meet the unique needs of users, processes for engaging users early and sustaining that engagement over time continue to be a high priority to fully realize the potential of technology-based ART adherence interventions. Finally, efforts should be made to expand our understanding of how peer exchange forum use may have positive impacts across a range of clinical and mental health domains.

## Appendix

Multimedia Appendix 1 Example newsletter received by participants randomized to the control group.

Multimedia Appendix 2 The impact user engagement in the Thrive with Me intervention on HIV undetectable viral load.

Multimedia Appendix 3 CONSORT-eHEALTH checklist (V 1.6.1).

## Abbreviations

ART: antiretroviral therapy
CONSORT: Consolidated Standards of Reporting Trials
DD: difference-in-differences
IMB: Information-Motivation-Behavioral Skills
mHealth: mobile health
RD: risk difference
THC: tetrahydrocannabinol
TWM: Thrive with Me
UVL: undetectable viral load
VL: viral load

## Data Availability

The data sets generated and analyzed during this study are available from the corresponding author on reasonable request.
