# Supplementary material for: A Web-Based Antiretroviral Therapy Adherence Intervention (Thrive With Me) in a Community-Recruited Sample of Sexual Minority Men Living With HIV: Results of a Randomized Controlled Study
Source: J Med Internet Res. 2024 Sep 30;26:e53819. doi: 10.2196/53819 (PMC11474139; doi:10.2196/53819)
Supplement: Multimedia Appendix 1 [file jmir_v26i1e53819_app1.pdf]

HIV and Stress Management:

CONQUERING STRESS

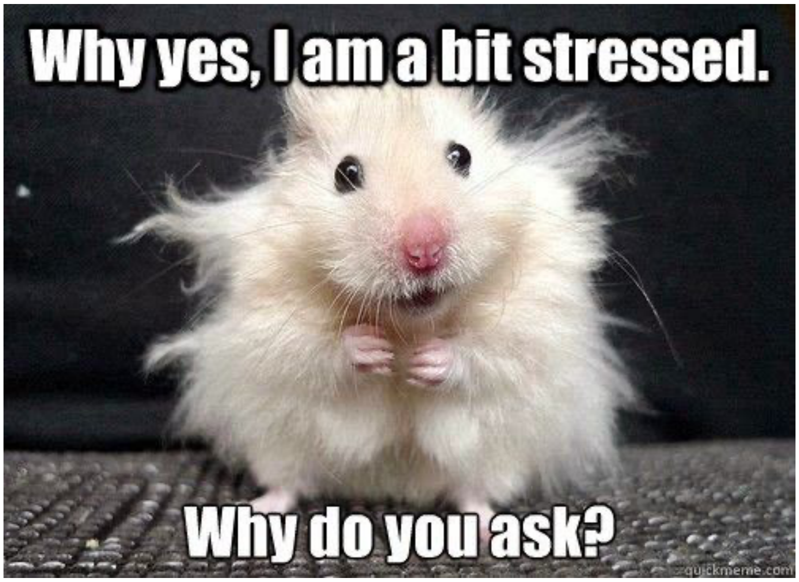

Stress is a feeling of tension, worry, or frustration, and it impacts us physically as well as emotionally. There are many common stressors that we encounter every day without even realizing it. Stress can be caused by different things for different people, but either way it's hard to avoid.

Research shows that ongoing stress can have a negative impact on your body-- affecting your sleep, mood, sex drive, appetite, digestion, and your immune system, to name a few.

Everyone deals with some amount of stress in their daily life. But for someone who is HIV positive, stress can be heightened by managing healthcare, strains on interpersonal relationships, medication side effects, and more. Chronic stress can make you more susceptible to viruses and infections, while increasing the amount of time it takes to recover.

Instead of avoiding stress, learn to manage it. If you are the type of person who likes to manage stress by making lists, this may work for you:

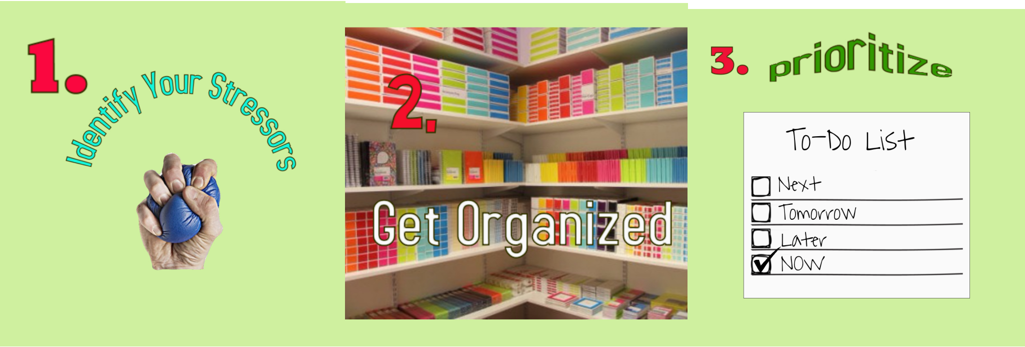

Chronic stress and anxiety can feel debilitating and isolating. Sometimes it can feel impossible to give yourself a break. Remember that you are not alone--it's okay to ask for help. See below for some tips on easy ways to give yourself a time out!

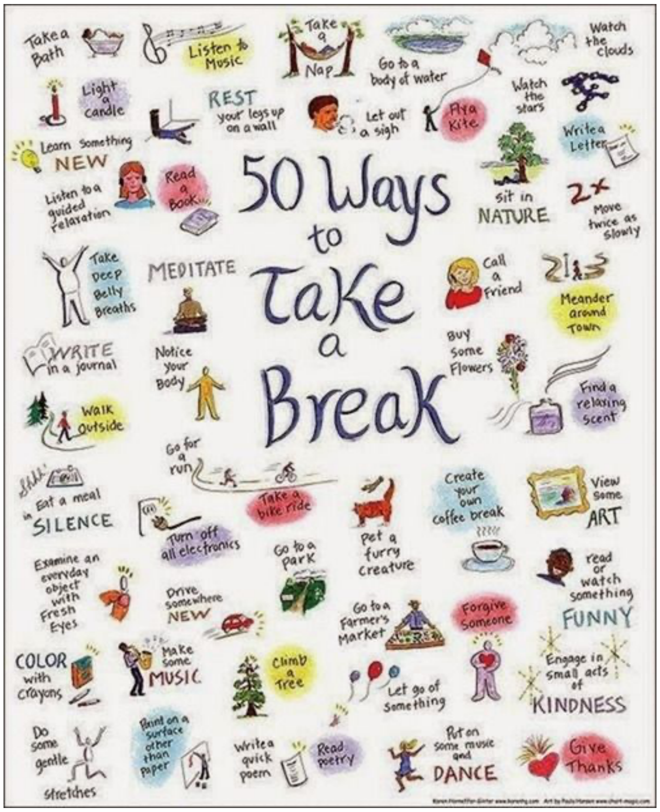

Image credit @Karen Horneffer-Ginter and Paula Hansen

Looking for more advice on how to manage stress? Click here!
