# Supplementary material for: A Web-Based Antiretroviral Therapy Adherence Intervention (Thrive With Me) in a Community-Recruited Sample of Sexual Minority Men Living With HIV: Results of a Randomized Controlled Study
Source: J Med Internet Res. 2024 Sep 30;26:e53819. doi: 10.2196/53819 (PMC11474139; doi:10.2196/53819)
Supplement: Multimedia Appendix 2 [file jmir_v26i1e53819_app2.pdf]

Multimedia Appendix 2. The impact user engagement in the Thrive with Me (TWM) intervention on HIV undetectable viral load (UVL).

Variable	TWM intervention (n=202), n (%)	UVL, n (%)	Risk difference (95% CI)[a]	Risk difference (95% CI)[b]
**<u>Month 5</u>**				
**Overall engagement[c]**				
High engagement	26 (12.9)	19 (73.1)[d]	*31.9 (12.6 to 51.3)*[e]	*19.4 (3.3 to 35.5)*[e]
Low engagement	84 (41.6)	45 (53.6)[f]	8.5 (−7.4 to 24.5)	1.6 (−12.7 to 16.0)
Nonusers (reference)	92 (45.5)	37 (40.2)[g]	Reference	Reference
**Peer exchanges**				
High engagement	27 (13.4)	19 (70.4)[h]	*25.3 (4.9 to 45.7)*[e]	14.6 (−2.9 to 32.2)
Low engagement	83 (41.1)	45 (54.2)[i]	10.1 (−5.9 to 26.1)	2.7 (−11.6 to 17.1)
Nonusers (reference)	92 (45.5)	37 (40.2)[g]	Reference	Reference
**Unique Thrive Tips[j]**				
High engagement	25 (12.4)	17 (68)[k]	19.0 (−2.0 to 40.0)	7.2 (−11.2 to 25.7)
Low engagement	75 (37.1)	39 (52)[l]	3.3 (−12.7 to 19.4)	−5.0 (−19.5 to 9.5)
Nonusers (reference)	102 (50.5)	45 (44.1)[m]	Reference	Reference
**ART[n] adherence self-monitoring**				

High engagement	26 (12.9)	16 (61.5)[d]	15.4 (−6.7 to 37.4)	4.4 (−17.1 to 25.8)
Low engagement	79 (39.1)	46 (58.2)[o]	12.6 (−3.3 to 28.4)	4.5 (−9.5 to 18.4)
Nonusers (reference)	97 (48)	39 (40.2)[p]	Reference	Reference
**Month 11**				
**Overall engagement[c]**				
High engagement	26 (12.9)	16 (61.5)[d]	20.3 (−2.2 to 45.8)	6.8 (−13.0 to 26.7)
Low engagement	84 (41.6)	41 (48.8)[f]	4.5 (−12.5 to 21.5)	−0.6 (−15.3 to 14.0)
Nonusers (reference)	92 (45.5)	32 (34.8)[g]	Reference	Reference
**Peer exchanges**				
High engagement	27 (13.4)	18 (66.7)[h]	*22.5 (1.1 to 44.0)[e]*	7.7 (−11.8 to 27.2)
Low engagement	83 (41.1)	39 (47)[i]	3.3 (−13.9 to 20.4)	−1.0 (−15.7 to 13.8)
Nonusers (reference)	92 (45.5)	32 (34.8)[g]	Reference	Reference
**Unique Thrive Tips[j]**				
High engagement	25 (12.4)	15 (60)[k]	13.9 (−8.9 to 36.7)	2.1 (−15.1 to 19.4)
Low engagement	75 (37.1)	36 (48)[l]	2.9 (−14.1 to 19.8)	−5.2 (−21.2 to 10.8)
Nonusers (reference)	102 (50.5)	38 (37.3)[m]	Reference	Reference

**ART adherence self-monitoring**				
High engagement	26 (12.9)	13 (50)[d]	3.4 (−20.3 to 27.1)	−6.7 (−29.8 to 16.4)
Low engagement	79 (39.1)	42 (53.2)[o]	8.6 (−20.3 to 27.1)	2.3 (−11.9 to 16.5)
Nonusers (reference)	97 (48)	34 (35.1)[p]	Reference	Reference
**Month 17**				
**Overall engagement[c]**				
High engagement	26 (12.9)	16 (61.5)[d]	21.2 (−1.2 to 43.5)	13.7 (−7.6 to 35.1)
Low engagement	84 (41.6)	38 (45.2)[f]	1.2 (−15.7 to 18.1)	−2.7 (−18.3 to 13.0)
Nonusers (reference)	92 (45.5)	33 (35.9)[g]	Reference	Reference
**Peer exchanges**				
High engagement	27 (13.4)	18 (66.7)[h]	*23.4 (2.2 to 44.7)[e]*	14.7 (−6.1 to 35.6)
Low engagement	83 (41.1)	36 (43.4)[i]	-0.1 (−17.1 to 16.9)	−3.1 (−18.8 to 12.6)
Nonusers (reference)	92 (45.5)	33 (35.9)[g]	Reference	Reference
**Unique Thrive Tips[j]**				
High engagement	25 (12.4)	15 (60)[k]	13.4 (−9.2 to 36)	5.3 (−14.5 to 25)
Low engagement	75 (37.1)	32 (42.7)[l]	-4 (−20.7 to 12.9)	−12 (−28.5 to 4.4)

Nonusers (reference)	102 (50.5)	40 (39.2)[m]	Reference	Reference
**ART adherence self-monitoring**				
High engagement	26 (12.9)	13 (50)[d]	8.3 (−15.5 to 32.2)	1.1 (−23.4 to 25.6)
Low engagement	79 (39.1)	40 (50.6)[o]	7.2 (−9.5 to 24)	3.3 (−12 to 18.5)
Nonusers (reference)	97 (48)	34 (35.1)[p]	Reference	Reference

[a]Unadjusted models.

[b]Models adjusted for baseline viral suppression and positive drug urinalysis.

[c]Active days in the TWM intervention.

[d]N=26.

[e]Estimate were statistically significant at *P*<.05.

[f]N=84.

[g]N=92.

[h]N=27.

[i]N=83.

[j]Brief informational content.

[k]N=25.

[l]N=75.

[m]N=102.

[n]ART: antiretroviral therapy.

[o]N=79.

[p]N=97.
